# Application of an Amine Functionalized Biopolymer in the Colonic Delivery of Glycyrrhizin: A Design and In Vivo Efficacy Study

**DOI:** 10.3797/scipharm.1301-14

**Published:** 2013-05-18

**Authors:** Amit Kumar De, Sriparna Datta, Arup Mukherjee

**Affiliations:** Department of Chemical Technology, Division of Pharmaceuticals and Fine Chemical Technology, University College of Science and Technology, University of Calcutta, 92, A. P. C. Road, Kolkata 700 009, India.

**Keywords:** Biopolymer, Guar gum alkyl amine, Glycyrrhizic acid, Factorial design, Colonic delivery

## Abstract

In our current study, a newer amine functionalized guar gum derivative was studied for its efficacy in colonic drug delivery. Glycyrrhizic acid mono-ammonium salt was used as the model drug. Drug-loaded microparticles were formulated by ionic crosslinking using sodium tripolyphosphate. The Scanning Electron Microscopic study revealed spherical particles of sizes from 4.9 ± 3.8 μm to 6.9 ± 3.9 μm. The FT-IR studies presented a possible interaction between the drug and the polymer. The drug was encapsulated in amorphous form as observed from the powder X-Ray Diffraction studies. A cumulative drug release study was carried out in simulated gastric, intestinal, and colonic fluids. The cumulative drug release studies presented a burst release followed by a sustained release of the drug in simulated colonic fluid containing rat cecal contents. The drug-polymer ratio was optimised using a 3^2^ factorial design by taking the amounts of glycyrrhizic acid (X_1_) and guar gum alkyl amine (X_2_) as the independant variables. The percent cumulative drug release at 240 mins (Q_240_), 720 mins (Q_720_), and at 1,440 mins (Q_1440_) were considered as the dependant variables. The efficacy of the optimized formulation was studied in a 2,4,6-trinitrobenzene sulfonic acid-induced rat colitis model. The tissue’s nitric oxide, malondialdehyde, and myeloperoxidase activities were found to be much lower in the microparticle-treated group compared to free drug-treated group. The histology of the colonic tissue from the treated group of animals revealed almost no infiltration of inflammatory cells in the tissue for the microparticle-treated group of animals. The synthesized amine derivative of guar gum was found to be better *in vitro* with a better *in vivo* efficacy in the colonic delivery of glycyrrhizic acid monoammonium salt and can be considered as a newer modified biopolymer for colonic drug delivery.

## Introduction

Inflammatory bowel disease is a common disease among urban populations [1–4]. Drugs prescribed for the treatment of this disease manifest a large number of side effects. A localised delivery is therefore suitable for the treatment of this disease. The development of polymer-based microspheres, microcapsules, and microbeads are currently the focused area of research for intestinally targeted drug delivery [5]. This also prevents a sudden unwanted higher concentration of the drug in the tissue and maintains controlled release of the drug at the target site. Polysaccharides are mainly used in the design of oral drug delivery devices. The increased adhesion of particles in the thick mucosal zones of inflammation or ulceration is an effective physiological parameter that can be exploited for targeted delivery of the drug encapsulated in the microcarriers [5, 6]. In inflammatory bowel disease, microparticulate drug carriers are expected to accumulate at the sites of inflammation in the colon. Due to effective adhesion to colonic mucosa, these carriers will have enough time to deliver their drug load to the sites of inflammation in the colon. Guar gum obtained from guar flour is a polysaccharide commonly used for designing colon-targeted drug delivery devices [7, 8]. Guar gum is metabolised by the colonic microflora that releases its contents specifically to the colon. A newer amine derivative of guar gum has been synthesised from commercially available guar gum. The derivative retains the biological properties of the mother polysaccharide and shows additional properties of the amine group. This makes the polymer positively charged. In our study, this newly synthesised derivative was utilized for the preparation of microparticles. Preparation of guar gum microparticles by the ionic gelation technique using metal ions and glutaraldehyde has been reported earlier [9–11]. In our current study, we used sodium tripolyphosphate as the crosslinker with guar gum alkyl amine.

Glycyrrhizic acid is a triterpene saponin found from the root extract of Glycyrrhiza glabra [12]. Glycyrrhizin is found to have anti-inflammatory, antiulcer, anti-allergic, immune-modulating, and antiviral properties [13–17].

Colonic delivery of microencapsulated glycyrrhizic acid can be used to ameliorate the severity of colitis due to its anti-inflammatory [18, 19] properties and its efficacy in the amelioration of peptic ulcer and inflammatory bowel disease [20, 21]. In our study, glycyrrhizic acid monoammonium salt (GA) was used as the model drug. The prepared microparticles were characterised using the Scanning Electron Microscopy (SEM), Fourier Transform Infrared Spectroscopy (FT-IR), and Powder X-Ray Diffraction (PXRD) studies. The entrapment efficiency and *in vitro* drug release were studied in simulated gastric, intestinal, and colonic fluids containing rat cecal contents. The drug-polymer ratio was optimized using a 3^2^ factorial design model. The *in vivo* efficacy of the microparticles was studied in a 2,4,6-trinitrobenzene sulfonic acid-induced rat model of colitis. The efficacy of the formulations was determined following a clinical score system [22].

An increase in the number of neutrophils, natural killer cells, mast cells, and regulatory T-cells has been reported to play an important role in the pathophysiology of inflammatory bowel disease [23]. Myeloperoxidase (MPO) is an important constituent of inflammatory cells, mainly the neutrophils [24]. The estimation of tissue MPO-content could be an efficient tool to measure the extent of colonic inflammation. In TNBS-induced colitis, the tissue NO was found to increase [25, 26]. Malondialdehyde (MDA), a secondary product of lipid peroxidation, was considered as an important parameter to study the extent of tissue damage [27].

Analysis of all three inflammatory parameters was carried out to study the efficacy of the GA microparticles in the amelioration of colonic ulcer. The results were corroborated by the histological studies of the colon tissue of the normal, control, and treated groups of animals.

## Experimental

### Materials

Glycyrrhizic acid monoammonium salt (purity = 98.1%) (glycyrrhizin; GA), sodium tripolyphosphate (TPP), and dialysis tubing D9652 (MW cut off 12,400 kD) were purchased from Sigma Aldrich India Ltd. Guar gum (mannose to galactose ratio 1:2) and all other reagents used for the synthesis of the alkyl amine derivatives were purchased from Merck India Ltd. Phosphoric acid of HPLC quality and the solvents used for chromatography of HPLC grade were purchased from Spectrochem India Limited.

Male Sprague-Dawley rats (6 weeks old, 190–210g) were purchased from the Central Ayurvedic Research Institute (Kolkata, India) and kept on a normal diet with water *ad libitum*. The animals were housed in polymer cages at 25 ± 1ºC temperature, 60 ± 5% relative humidity, and a 12-hour light-dark cycle. They were acclimatized for seven days before any treatment.

### Synthesis of Guar Gum Alkyl Amine

Guar gum alkyl amine (GGAA) was synthesised from guar gum following a single-step method mentioned elsewhere [28]. The product was washed several times with an isopropanol-water mixture in order to remove unwanted chemicals and reagents. The product was air-dried. The final product was purified by dialysis against Millipore water for 48 hours.

### Preparation of Microparticles (GAMs)

An aliquot of 0.1% GGAA solution was taken in a glass homogeniser and homogenised at 8000 rpm on an ice bath. A 0.1% GA solution was added dropwise to the GGAA solution under homogenisation in an ice bath followed by 0.1% TPP solution under similar conditions. The homogenisation was further continued for 30 minutes in an ice bath and thereafter Tween 80 (0.036% solution) solution was added dropwise and homogenised. The product was stirred overnight using a magnetic stirrer. The final product was harvested by centrifugation at 10,000rpm for 15 minutes at 4°C, washed three times with HPLC grade water, dried for 3 days in a vacuum desiccator, and preserved at 4°C in tightly closed vials till further use.

### Characterization of GAMs

The GAMs were characterized by their shape and particle size. A Scanning Electron Microscope (SEM) (Ion Sputter S-3400N Hitachi, Japan) was used for the physical characterization of the GAMs. The sample was analysed at 15kV at 4.50k SE magnification. The particle size was determined by measuring the diameters of 100 particles chosen at random. The particle shape and surface were examined using the SEM photomicrographs.

The FT-IR spectra for GA, GGAA, and the GAMs were recorded in Spectrum One FT-IR spectrometer (Perkin Elmer, USA) in pressed potassium bromide pellets. The spectra were stacked for the determination of any drug-polymer interactions.

The X-ray diffraction patterns were determined with the X-ray powder diffractometer (XPERT-PRO, PANalytical) using CuKα radiation at a scan rate of 0.0330°/15.0479sec over the 2θ range of 5–90°. Diffraction patterns of the pure drug, polymers, physical mixture, and the prepared microparticles were compared.

#### Entrapment Efficiency

A validated reversed-phase chromatographic method was used throughout. The chromatographic determinations were carried out on a Waters (Waters, USA) binary gradient system equipped with a Waters 515 pump (two numbers), a manual Rheodyne injector port attached with a 20μl loop, and a Waters 2996 PDA detector. The system control and data acquisition were carried out using Empower 2 software (Waters, USA). The separation was carried out in a reversed-phase Kromasil C18 column (125mm × 4.0mm, 5μ; Akzonobel, USA). The standard, sample, and mobile phase (mixture of 35% acetonitrile and 65% phosphate buffer (pH = 3.0), prepared by dissolving potassium phosphate monobasic 2.1012g and potassium phosphate dibasic 1.0852g in 1000ml water, adjusted to pH=3.0 using orthophosphoric acid) was filtered through a 0.2μm membrane filter (Pall Life Science, India). The flow rate was 1.0 ml/minute and the column was maintained at ambient temperature. The column effluent was monitored at 252nm with PDA detection. The retention time of GA was 8.5 ± 0.09 minutes (± S.D.; n=3). A peak area (along y axis) vs. concentration in μg/ml (along × axis) graph for GA was first prepared (y = 21493× + 22074, *R^2^= 0.9995*) and used to estimate GA concentrations throughout. The mass of GA in solution before and after the microparticulation in supernatant was determined by the HPLC experiments for the calculation of entrapment efficiencies as presented in Equation 1.

Eq. 1 $$\text{GA }\%\;\text{encapsulation}=\frac{\left(\text{Mass of GA originally taken}-\text{Mass}\;\text{of GA in supernatant}\right)}{\left(\text{Mass of GA originally taken}\right)}\times 100$$

#### Preparation of 4% Rat Cecal Content Medium

Male Sprague-Dawley (SD) rats weighing between 125 to 150 gm were used in the test. The protocol was approved by the Institutional Animal Ethics Committee (IAEC), University of Calcutta, Department of Chemical Technology with CPCSEA registration number 506/01/a/CPCSEA. The rats were asphyxiated by excessive inhalation of carbon dioxide (CO_2_) [29]. The abdomens were opened and the cecum were traced, legated at both ends, dissected, and immediately transferred to the simulated colonic fluid [8] previously bubbled with CO_2_. The cecums were opened, weighed separately, pooled, and then a 4% solution was prepared. The solution was then centrifuged at 5000 rpm for 15 minutes. The supernatant was used as rat cecal content medium in the dissolution studies.

#### In vitro Drug Release Study

**Fig. 1:**
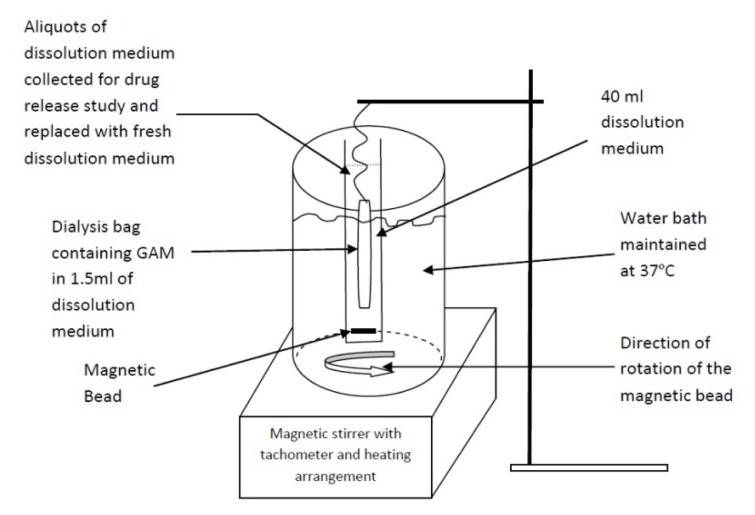
Pictorial representation of *in vitro* dissolution study

The *in vitro* drug release study was carried out in simulated gastric fluid of pH 1.2, simulated intestinal fluid of pH 6.8, and simulated colonic fluid of pH 7.4 containing rat cecal contents under anaerobic conditions. GAMs equivalent to about 10 mg of the drug GA were dispersed in 1.5 ml of the simulated biological fluids placed in dialysis bags and both ends were sealed with a clean thread. The bag was suspended in 40ml of the respective simulated fluid taken in a tube and maintained at 37°C in a beaker under constant stirring at 50rpm (Fig. 1). At predetermined time intervals, the release medium from the tube was taken out and replaced with fresh dissolution medium. The amount of GA released at regular intervals was estimated by the HPLC method mentioned under the drug entrapment study.

#### Factorial Design and Optimization of the Drug-Polymer Ratio

A 3^2^ (three level two factors) full-fledged factorial design [30] was applied to optimize the two independent variables, namely the amount of GA (X_1_) and the amount of GGAA (X_2_). The factorial design layout for the nine different batches are presented in Tab. 1A and 1B. The *in vitro* drug release in the simulated colonic fluid was monitored first at 30mins and then every hour. The release at 240mins, 720mins, and 1,440mins were analyzed as response parameters in the factorial design studies. Results are expressed as the second order polynomial Equation 2.

Eq. 2 $${\text{Y}}_{\text{i}}={\text{b}}_{0}+{\text{b}}_{1}{\text{X}}_{1}+{\text{b}}_{2}{\text{X}}_{2}+{\text{b}}_{12}{\text{X}}_{1}{\text{X}}_{2}+{\text{b}}_{11}{{\text{X}}_{1}}^{2}+{\text{b}}_{22}{{\text{X}}_{2}}^{2}$$

Where b_0_ denotes the arithmetic mean response for nine runs, b_i_ (i = 1, 2) denotes the estimated coefficient for the factors and X_i_ (i = 1, 2) denotes the effect of changing one factor at a time from its lowest to highest level. The interaction terms X_1_X_2_ denotes the effect when both the factors were changed simultaneously. The polynomial terms X_i_^2^ (i = 1, 2) was used to explain non-linearity [31]. Y was the measured response parameter in each experiment and was taken as a dependant variable. The coefficients corresponding to the linear effects, b_1_ and b_2_, interactions, b_12,_ and the quadratic effects, b_11_ and b_22,_ were determined from the experimental results.

#### Colitis Model

**Fig. 2:**
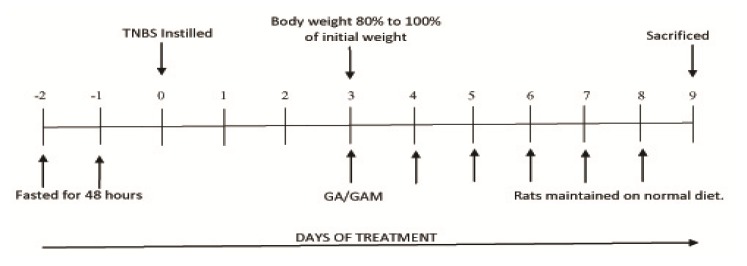
Programme schedule for the development of experimental colitis and subsequent drug treatment.

All animal experiments were performed in accordance with the recommendations of the Institutional Animal Ethics Committee, Dept. of Chemical Technology, University of Calcutta, India with registration number 506/01/a CPCSEA. The TNBS rat model is a recognized model for the induction of experimental colitis with a very low mortality rate [32]. The inflammation was induced in male SD rats by instilling 0.85 ml of the TNBS-ethanol mixture (0.6ml of 5% w/v TNBS in 0.25ml of 50% ethanol) into the lumen of the colon using a baby feeding tube, followed up by flushing the tube with 0.5ml of air. The treatment schedule is presented in the figure below (Fig. 2).

The animals in the treatment group received either 1.0ml of the suspension of GAM in saline or GAM at a dose equivalent to 50mg of GAM per kg body weight once daily. The TNBS control group received only saline and the void control group received the void microparticle suspension. The animals were sacrificed 24 hours after administration of the last drug/GAM dose. Their abdomens were cut open and the colon was legated at both ends. Finally, the colon was resected, cleaned, and preserved till further use.

#### Pathophysiological Parameters

The degree of severity of colitis and inflammation was analyzed using a clinical activity index like weight loss, stool consistency, and rectal bleeding as described earlier [22].

The resected colon tissue was opened longitudinally and cleaned thoroughly with ice cold phosphate buffer saline to remove its luminal contents. The colons were examined for the visual severity of the colitis. The colon weight and length were examined. They were expressed as a ratio with the respective body weights. The measurement of nitric oxide and myeloperoxidase activity in the inflamed tissue was performed to study the extent of the severity of the colitis. The level of malondialdehyde in the colonic tissue was determined as an indicator of lipid peroxidation [22, 24, 25].

The other part of the colon tissue was preserved in formalin buffer solution for histological examination.

### Statistical Analysis

The results were presented as the mean ± S.D. values. For analysis of statistical significance, Student’s–*t* test and ANOVA were applied. Furthermore, a p<0.05 was considered to be significant.

## Results and Discussion

### Particle Characteristics

GGAA was synthesised, purified, and subjected to IR Spectroscopic analysis for the presence of an amine group. The dried purified product was used for the preparation of GAMs. The GAMs were prepared by ionic crosslinking by using sodium tripolyphosphate.

**Fig. 3:**
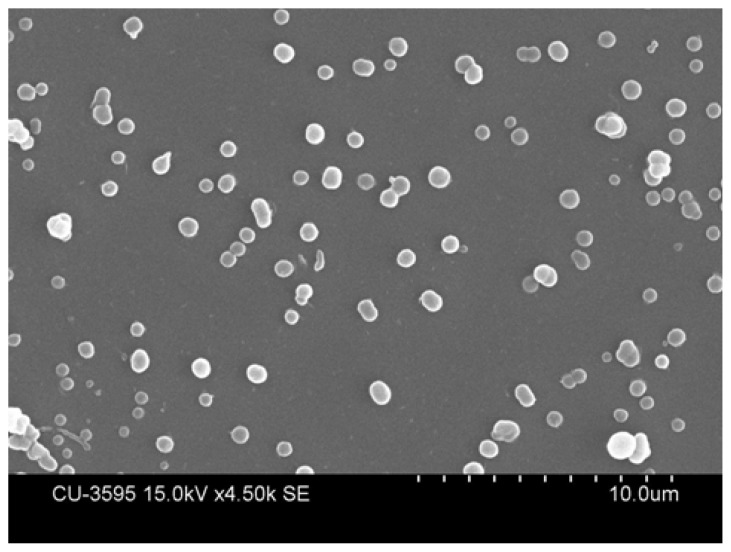
Representative scanning electron photomicrograph of prepared microparticles

The microparticles were almost spherical with an average particle size varying from 4.9 ± 3.8μm to 6.9 ± 3.9 μm (Tab. 1A) as revealed from scanning electron photomicrograph (Fig. 3). Studies by Lampretch et al. and his colleagues have shown that the bioadhesion of the microparticle carriers to the inflamed colonic mucosa depends on the size of the microparticle [5]. Also, particle sizes smaller than 200μm were found to show prolonged passage time. This indicates a longer residence time for the prepared microparticles in the colon for the delivery of the total drug load into the colonic mucosa. The prepared microparticles also had smooth surfaces with very few aggregations.

#### FT-IR Spectroscopy

**Fig. 4:**
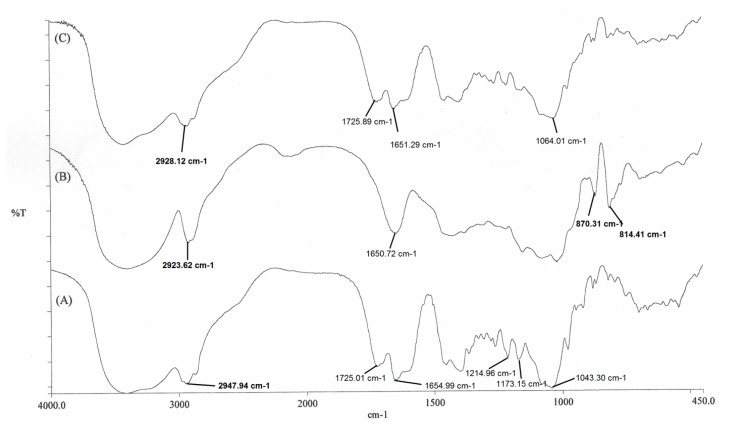
FT-IR spectrums of pure Glycyrrhizic Acid monoammonium salt (A), Guar Gum Alkyl Amine (B), microparticle formulation (C)

The FT-IR spectroscopy (Fig. 4) of the optimized formulation F4 was carried out to observe any drug-polymer interaction. GA showed a sharp peak at 1725.01cm^−1^ which showed no shift in the GAM formulations. However, a sharp shift was observed from 1043.3cm^−1^ in the GA spectrum to 1064 cm^−1^ in the formulation.

The peak at 1043.3cm^−1^ in the GA spectrum might be due to a primary alcohol and its shift to 1064 cm^−1^ was due to a hydrogen bonding interaction between GA and GGAA [33]. This might be the reason for satisfactory entrapment of GA within the GAMs.

#### Powder XRD Studies

**Fig. 5:**
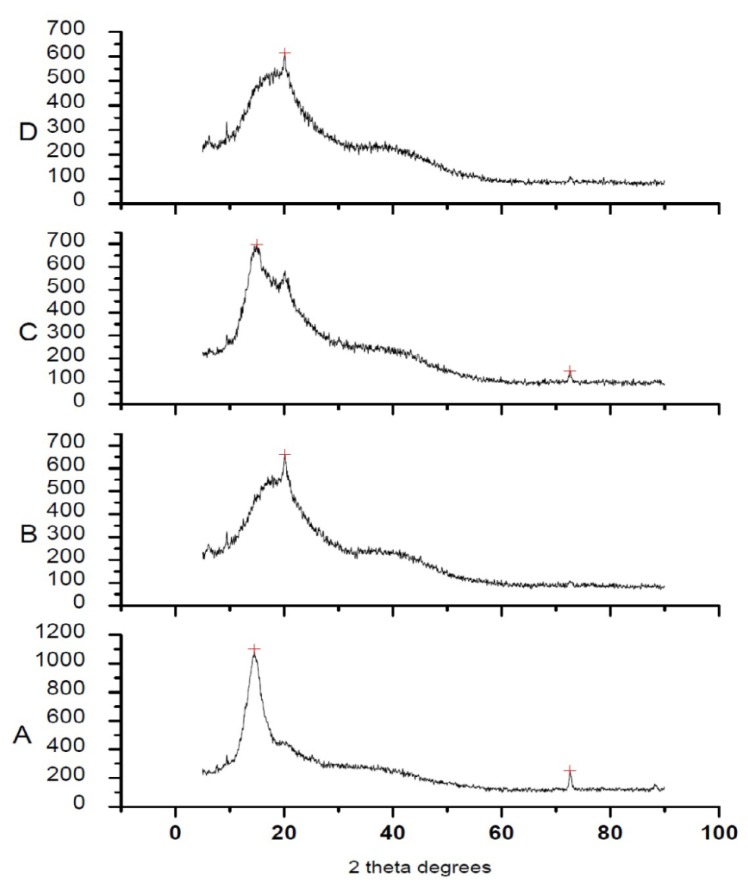
Powder XRD of pure GA (A), GGAA(B), physical mixture (C), GAM (D)

The X-ray diffractograms (Fig. 5) manifested two distinct peaks at 2θ: 14.65° and 72.63° for intact GA and a clear peak at 20.19º for GGAA. The diffraction pattern of the physical mixtures presented the lowering in peak intensities of the intact drug due to the dilution effect of the polymer and no qualitative differences in the diffraction pattern of the drug. GAMs prepared with GGAA were characterized by the absence of the distinct diffraction peaks of GA and a peak for the polymer at 20.29°, signifying the entrapment of the drug in amorphous form or its solvation in the polymeric carrier (Fig. 5). Therefore, the PXRD studies verified the decrease in crystallinity of the drug in the microparticles and the effective entrapment of GA in amorphous form within the polymer matrix.

#### Drug Entrapment Efficiency

The drug entrapment within the GAMs was found to be on the higher side for all the nine formulations F1 to F9. The average drug entrapment varied from 75.36 ± 5.69% to 98.36 ± 4.65% of the total drug used for each GAM formulation (Tab. 1A, 1B).

#### In vitro Drug Release

**Fig. 6:**
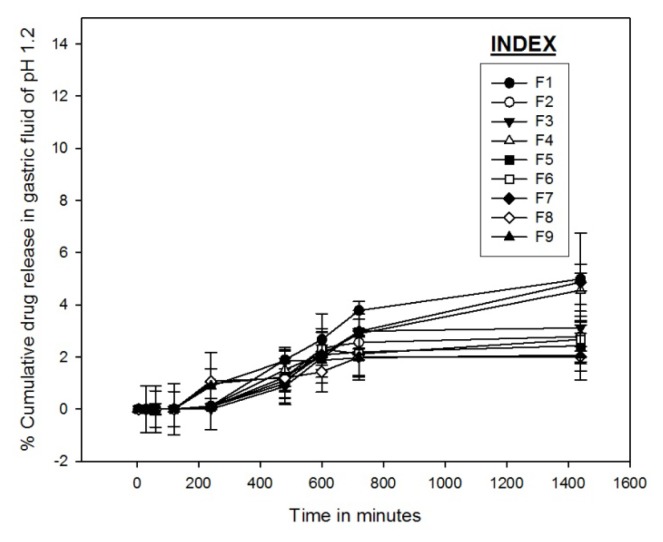
Cumulative drug release profile of nine formulations in simulated gastric fluid

**Fig. 7:**
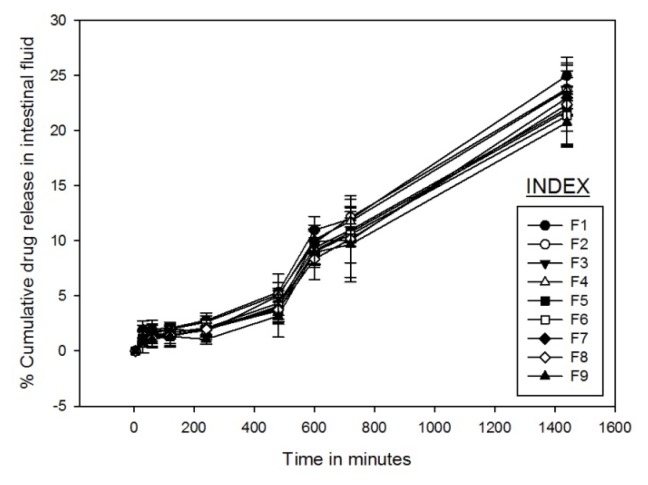
Cumulative drug release profile of nine formulations in simulated intestinal fluid

The drug release studies from the microparticles were carried out in the simulated gastric fluid of pH 1.2, simulated intestinal fluid of pH 6.8, and simulated colonic fluid of pH 7.4 containing rat cecal contents [34, 35]. Altogether, nine formulations F1 to F9 were prepared with varying proportions of the polymer and the amount of drug. Fig. 6, 7, and 8 demonstrate GA release profiles from GAMs in three different mediums of pH 1.2, 6.8, and 7.4 respectively. In the simulated gastric and intestinal fluid, minimum drug release was observed and the rate of drug release was also very slow. In the simulated gastric fluid for formulations F1, F4, and F7, a maximum of only 5% cumulative drug release was observed, whereas for the others it was only about 2%. In the simulated intestinal fluid, only about 10% of the drug was released in the first 600 mins of the study for all nine formulations. In the simulated colonic fluid containing rat cecal contents, the drug release was a bit more augmented with an initial burst release common for each of the designed formulations, followed by a sustained release of GA. It was observed that for all nine formulations, after 240 minutes, the percentage cumulative drug release varied from 43.5±2.56% to 70.4±2.36%; after 720 minutes it varied from 76.9±3.75% to 93.9±2.45%; and after 1440 mins of the study it varied from 82.9±1.65% to 95.8±1.23% in the simulated colonic fluid.

The results predict the drug–polymer ratio to be the key factor for controlling the release. However, a complex interaction between the drug and polymer molecules or the simple adsorption of the drug molecules on the surface of the polymeric MPs due to electrostatic adhesions may be responsible for the successful entrapment of the drug within the polymer matrix.

A minimum drug release in the simulated gastric and intestinal fluid implies a minimal chance of the drug molecule getting adsorbed to the surface of the microparticle, as adsorption of GA on the surface of the microparticle would result in a substantial drug release earlier in the simulated gastric and intestinal fluid. Thus, a proper entrapment of GA might be predicted from the study. The increase in drug release in the simulated colonic fluid was facilitated by the enzymatic degradation of the polymer and by the colonic microflora present in the rat cecal contents. The nature of the release profile predicts the dependence of the cumulative drug release on the drug-polymer ratio. Therefore, the formulation was optimized on the basis of the drug-polymer ratio using a 3^2^ factorial design model.

#### Factorial design and optimization

The response obtained from in vitro release studies was evaluated using a statistical model which involved a number of polynomial terms as explained earlier. The percent cumulative drug release values at three different time intervals of all nine formulations were fitted in the quadratic equation 2. The coefficients were determined for responses Q_240_, Q_720_ and Q_1440_ and the respective equations 3, 4 and 5 were obtained. The data indicated that the release profile of the drug was strongly dependant on the selected independent variables i.e the drug-polymer ratio. The data fitted in equation for the full model relating to responses Q_240_, Q_720_ and Q_1440_ and the transformed factors have been presented in Tab 2. The resultant equation for all three dependent variables Q_240_, Q_720_ and Q_1440_ – in terms of their coded factors were as follows:

Eq. 3 $${\text{Q}}_{240}=+57.23-3.55{\text{X}}_{1}-9.95{\text{X}}_{2}-2.42{\text{X}}_{1}{\text{X}}_{2}+0.65{{\text{X}}_{1}}^{2}+1.35{{\text{X}}_{2}}^{2}$$

Eq. 4 $${\text{Q}}_{720}=+88.62-1.88{\text{X}}_{1}-5.62{\text{X}}_{2}-0.85{\text{X}}_{1}{\text{X}}_{2}+0.62{{\text{X}}_{1}}^{2}-2.68{{\text{X}}_{2}}^{2}$$

Eq. 5 $${\text{Q}}_{1440}=+92.25-2.42{\text{X}}_{1}-3.72{\text{X}}_{2}-0.65{\text{X}}_{1}{\text{X}}_{2}+1.12{{\text{X}}_{1}}^{2}-2.38{{\text{X}}_{2}}^{2}$$

A positive value indicates a synergistic effect that favours optimization, while a negative sign represents an antagonistic effect or an inverse effect of the factor on the selected response. It was observed that for all the three dependant variables Q_240_, Q_720_ and Q_1440_, the linear contribution of both coded factors shows an antagonistic effect (p < 0.01, Tab. 2). For response Q_240_ the linear contribution of X_1_, X_2_ and the contribution of the interaction term X_1_X_2_ were significant with p < 0.01 respectively. At 720 mins (Q_720_) a significant contribution of the polymer in drug release from the formulations (p < 0.01) was observed. At 1440 minutes (Q_1440_) the individual contribution of drug and polymer became significant.

**Fig. 9:**
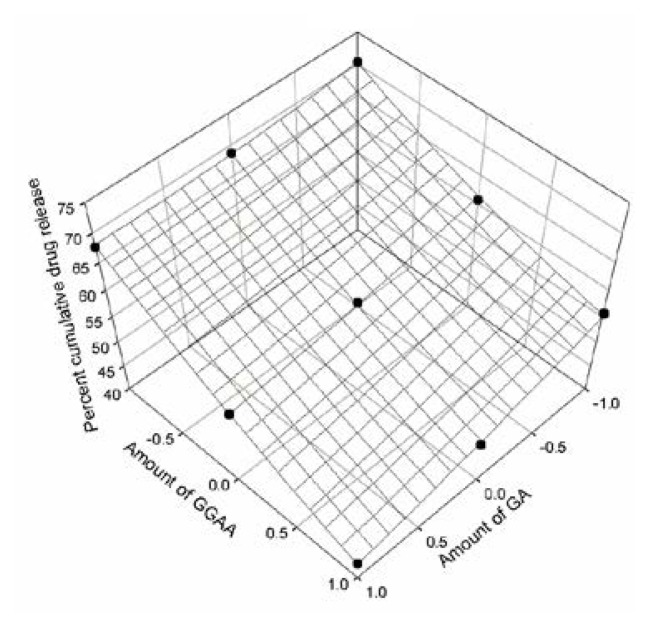
Response surface plot at 240 minutes of cumulative drug release study

**Fig. 10:**
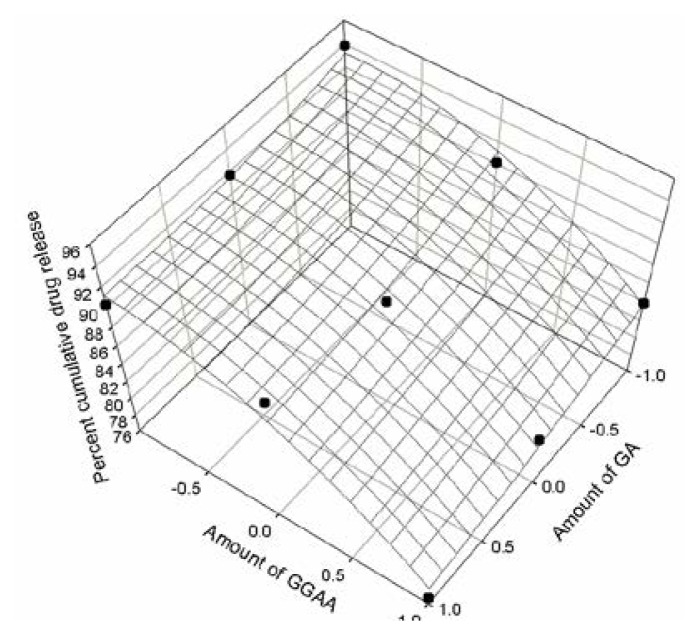
Response surface plot at 720 minutes of cumulative drug release study

**Fig. 11:**
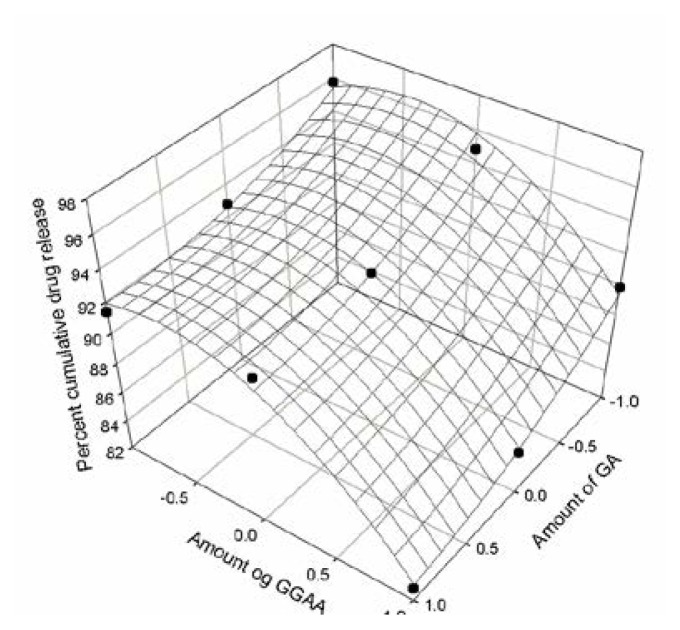
Response surface plot at 1440 minutes of cumulative drug release study

In order to determine the significance of each factor, analysis of variance was performed. The results have been presented in Tab. 3. The high values of correlation coefficients for Q_240_, Q_720_ and Q_1440_ indicated a good fit. The response surface regression analysis was performed using coded values of factor levels (−1, 0, +1) for each factor to understand the contribution of each independent variable. The response surface plots were presented in Fig. 9, 10 and 11 corresponding to release at 240 minutes, 720 minutes and 1440 minutes respectively.

The three dimensional plots gives us an idea about the change in the response surface.

The formulation was optimized on the basis of observed and predicted values of the responses [30, 31]. The observed values for the formulation F4 were very close to the predicted value. This implies that the cumulative drug release from formulation F4 followed the required pattern of drug release to the colon. The composition of optimized formulation F4 was GGAA 83.33% and GA 16.67% (Tab. 4).

#### In-vivo efficacy study

**Fig. 12:**
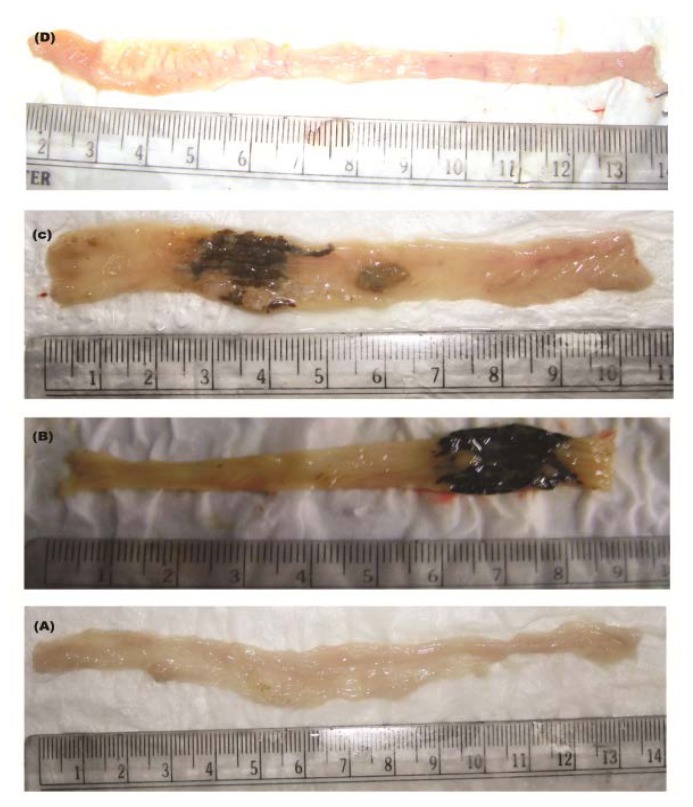
Longitudinal section of colon of normal rat (A), TNBS treated control rat (B), GA treated rat (C), and GAM treated rat (D).

The in vivo efficacy study was carried out with the optimized formulation F4 having drug/polymer ratio 2:10. The therapeutic efficacy of this optimized GAM formulation was studied on TNBS induced rat model of colitis. The animals under study were divided into six groups – normal control, solvent control, TNBS control, Void control, GA and GAM groups. The normal control group was kept to study the normal condition of colon. The solvent control group was kept to study whether the solvent used had any adverse effect. The void control group was kept in order to study the possible effect of the delivery device itself. The ameliorative efficacy of the GAM was studied by comparing the results with the GA treated groups. Intra-colonic administration of TNBS in 50% ethanol resulted in an inflammatory response characterized by extensive mucosal disruption, linear and deep ulcers, haemorrhage, and sub-mucosal oedema (Fig. 12). Diarrhoea and rectal bleeding were evident in all the rats with colitis which were without any treatment.

The results of the control groups were compared with the treated groups i.e. GA and GAM treated groups. Both the diarrhoea and the rectal bleeding was reduced significantly in both the drug treated groups but optimized formulation F4 showed higher efficacy in the healing process than that of GA and the studied parameters for GAM treated group was close to the healthy control group [22].

The length of the colon from appendix to anus was measured and was found to be 13.5cm for the normal group, 8.8cm for the TNBS control group, 10.4cm for GA group and 12.0cm for GAM group. Thus both GA and GAM recovered the colon length from shortening which was more significant for GAM. The colon to body weight ratio for GAM group was 8.34 and was much lower than the TNBS control group 20.84 (Tab 5). The tissue biochemical parameters namely tissue nitric oxide, myeloperoxidase and malondialdehyde presented a much lower value for those treated with GA and GAM compared to the TNBS control group, GAM treated group showing the lowest value for all the three studied parameters (p<0.05) indicating the best healing effect shown by GAM formulation (Tab. 5) [24, 26]. Thus encapsulation of GA within the microparticles in formulation F4 showed greater efficacy in the amelioration of colitis.

Histological examination presented extensive ulcer formation and infiltration of inflammatory cells mostly neutrophils and florid ulcer formation with inflammation and necrosis among the TNBS control group. The GA treated group showed presence of inflammatory cells and moderate improvement with granulated tissue formation (Fig. 12). Only in the case of GAM group mild ulcer and minimum inflammation was observed with very low infiltration of the inflammatory cells.

**Fig. 13:**
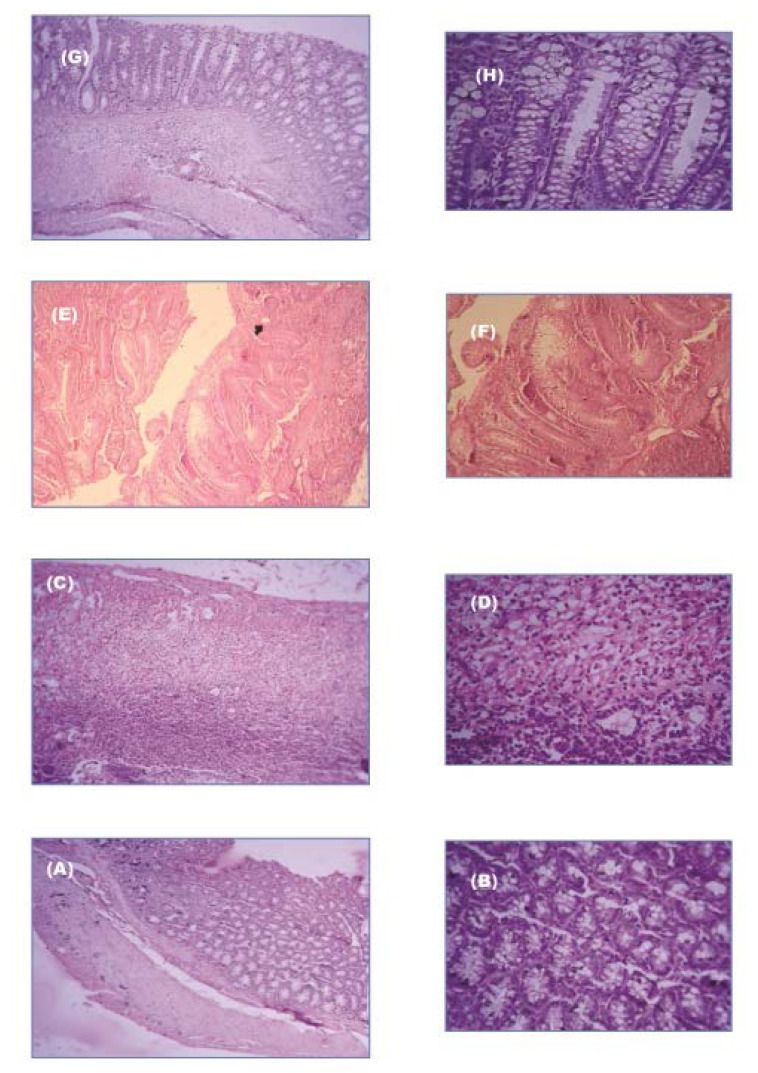
Haematoxylin and eosin stained paraffin section from colonic tissue. Normal colon at 10× magnification (A), normal colon at 40× magnification (B), TNBS treated colon at 10× magnification (C), at 40× magnification (D), GA treated colon at 10× magnification (E), at 40× magnification (F), GAM treated colon at 10× magnification (G), at 40× magnification (H)

The colon to body weight ratio (Tab. 5) and the colon length (Fig. 12) for the GAM treated group were close to healthy control group after treatment. The studied biological parameters (Tab. 5) present enhanced efficacy of GAMs over free GA [22]. The visual severity of colitis presented minimum damage of colonic mucosa in case of GAM treated animals compared with GA treated group (Fig. 12). Excessive accumulation of inflammatory cells mainly neutrophils was observed at the sites of inflammation in TNBS control group and an increased tissue myeloperoxidase [29] was thus observed. In case of GAM group, the MPO activity in the colonic tissue was found to be the minimum, indicating lesser inflammation and better healing of colitis [36, 37]. The histological examination of the colonic tissue (Fig. 13) presented least accumulation of inflammatory cells in colonic tissue of GAM treated animals [38, 39]. The higher localized delivery of the drug to the affected colonic tissue may have reduced the extent of ulcer formation compared to the GA treated group of animals where much of the drug may have been absorbed at normal tissues of intestine and colon.

## Conclusion

The monoammonium glycyrrhizinate-loaded microparticles prepared through the ionic crosslinking of a newer biopolymer guar gum alkyl amine. This formulation was found to deliver the optimal drug load to the colon. The FT-IR studies presented a possible interaction of the drug with the polymer and retention of the drug within the polymer matrix. The drug-polymer ratio was found to be 1:5 on the optimization using a 3^2^ factorial design model. The release profile of the optimised formulation and *in vivo* efficacy studies on the basis of physiological and biochemical parameters presented the successful application of this newer biopolymer in the preparation of colonic delivery devices.

## Figures and Tables

**Fig. 8:**
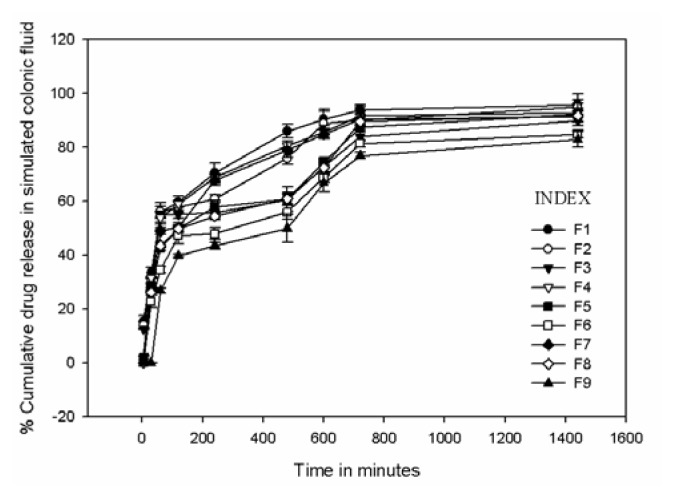
Cumulative drug release profile of nine formulations in simulated colonic fluid containing rat cecal contents under anaerobic condition

**Tab. 1A:** Formulation, drug-polymer ratio, drug entrapment, and cumulative drug release in simulated colonic fluid of nine formulations

Formulation	d/p ratio	X_1_^b^	X_2_^b^	Q_240_^c^ (%)	Q_720_^c^ (%)	Q_1440_^c^ (%)	% Drug entrapment^d^	Average particle size (μm)	Size distribution (min–max, μm)
F1	3:20	−1	−1	70.4 ± 2.36	93.9 ± 2.45	95.8 ± 1.23	98.36 ± 4.65	4.9 ± 3.8	3.8–8.6
F2^e^	3:30	−1	0	60.8 ± 2.95	90.2 ± 2.11	94.9 ± 1.87	95.63 ± 4.32	5.0 ± 2.7	4.7–6.9
F3	3:40	−1	1	55.8 ± 1.56	83.9 ± 2.39	89.9 ± 2.03	97.45 ± 1.23	6.9 ± 4.1	6.1–8.9
F4	2:10	0	−1	68.7 ± 2.09	91.7 ± 1.23	92.8 ± 1.11	96.41 ± 2.34	5.5 ± 1.2	5.0–7.9
F5	2:15	0	0	57.8 ± 1.98	87.4 ± 2.51	92.3 ± 1.01	94.56 ± 1.65	5.9 ± 2.9	5.2–8.0
F6^e^	2:20	0	1	47.9 ± 1.69	81.4 ± 1.98	84.9 ± 0.98	95.63 ± 3.65	6.5 ± 3.9	6.0–8.2
F7	1:4	1	−1	67.8 ± 3.69	90.3 ± 2.05	91.4 ± 2.22	75.36 ± 5.69	5.9 ± 6.1	4.8–9.5
F8	1:6	1	0	54.4 ± 2.99	89.5 ± 2.56	91.8 ± 2.58	82.36 ± 4.32	5.8 ± 5.2	5.0–7.5
F9	1:8	1	1	43.5 ± 2.56	76.9 ± 3.75	82.9 ± 1.65	80.98 ± 1.98	6.9 ± 3.9	6.1–9.2

b X_1_ indicates the amount of GA in mg and X_2_ the amount of GGAA in mg;

c Q_j_ denotes percent cumulative drug release at j ^th^ minutes with standard deviation values of three replicates expressed as a percentage of total drug load;

d Evaluated on the basis of mass of GA used at the time of preparation and the mass of GA found entrapped in each formulation;

e F2 and F6 were different formulations with variation in the amount of GA and GGAA used in mg, prepared for the interest of factorial design.

**Tab. 1B:** Amount of drug and polymer at different levels of factorial design study

Levels	GA(mg) [X_1_]	GGAA(mg) [X_2_]
−1	7.5	50
0	10	75
1	12.5	100

**Tab. 2:** Quantitative factor effects and the associated p values for all three responses

	Q_240_		Q_720_		Q_1440_	
	
Factor	Factor effects	p Value	Factor effects	p Value	Factor effects	p Value
X_1_	−3.5500	0.0014	−1.8833	0.0957	−2.4167	0.0071
X_2_	−9.9500	<0.0001	−5.6167	0.0056	−3.7167	0.0020
X_1_X_2_	−2.4250	0.0076	0.6167	0.4413	−0.6500	0.2437
X_1_X_1_	0.6500	0.3090	−2.6833	0.6806	1.1167	0.1770
X_2_X_2_	1.3500	0.0848	−0.8500	0.1427	−3.3833	0.0129

**Tab. 3:** Analysis of Variance

Responses	Regression	DF	SS	MS	R^2^	P
Q_240_	FM	5	697.6425	139.5285	0.9976	0.0004
	Residual					
	FM	3	1.6975	0.5658	–	–

Q_720_	FM	5	228.6144	45.7229	0.9538	0.0323
	Residual					
	FM	3	11.0678	3.6893	–	–

Q_1440_	FM	5	145.0011	29.0002	0.9836	0.0070
	Residual					
	FM	3	2.4211	0.8070	–	–

FM denotes full model.

**Tab. 4:** Optimization:

	Formulation F4		

Response	Observed	Predicted	Residual
Q_240_	68.70	68.53	0.17
Q_720_	91.70	91.55	0.15
Q_1440_	92.80	92.59	0.21

**Tab. 5:** *In vivo* efficacy study

	Colon to body weight ratio	Spleen to body weight ratio	NO nM/g of wet tissue	MDA nM/g of wet tissue	MPO U/min/g of wet tissue
Normal	5.01	4.41	51.63	13.01	0.11
TNBS	20.84	5.99	117.21	107.15	0.58
GA	11.97	4.06	77.80	35.89	0.34
GAM	8.34	4.18	61.07	25.58	0.24

## Abbreviations

GAM: Glycyrrhizic acid microparticles
GA: Glycyrrhizic acid mono ammonium salt
GGAA: Guar Gum Alkyl Amine
TNBS: 2,4,6-Trinitrobenzene Sulfonic Acid
UC: Ulcerative Colitis
CD: Crohn’s Disease
IBD: Inflammatory Bowel Disease
SEM: Scanning Electron Microscopy
FT-IR: Fourier Transform Infrared Spectroscopy
PXRD: Powder X-Ray Diffraction
SD: Sprague-Dawley
HPLC: High-Performance Liquid Chromatography
S.D.: Standard Deviation
CPCSEA: Committee for the Purpose of Control and Supervision on Experiments on Animals
IAEC: Institutional Animal Ethics Committee
MPO: Myeloperoxidase
MDA: Malodialdehyde
NO: Tissue Nitricoxide
TPP: Sodium tripolyphosphate
PDA: Photodiode Array
d/p ratio: Drug-polymer ratio
MPs: Microparticles
mins: minutesl
